# Decreased frontal theta frequency during the presence of smartphone among children: an EEG study

**DOI:** 10.1038/s41390-024-03155-x

**Published:** 2024-05-25

**Authors:** Rawnaq Shaer, Sheherban Nasser Eldin, Carmel Gashri, Tzipi Horowitz-Kraus

**Affiliations:** 1 Educational Neuroimaging Group, Faculty of Education in Science and Technology, Israel Institute of Technology, Haifa, Israel; 2 Kennedy Krieger Institute, Baltimore, MD USA; 3 Johns Hopkins University School of Medicine, Baltimore, MD USA

## Abstract

**Background:**

Smart devices have become an integral part of our lives. However, research has highlighted the potential implications of smartphone presence on task performance, particularly in young children. This study aimed to determine the effect of a smartphone presence on brainwaves associated with cognitive interruption in children.

**Methods:**

EEG data were collected from 5.3 to 8.5-year-old children performing a simple reaction time task with and without the presence of a smartphone. Theta and alpha bands were calculated, and repeated measure analysis of variance was performed to assess the impact of two conditions on alpha and theta bands: 1) with the presence and; 2) without the presence of a smartphone. EEG waveforms were also correlated with standardized cognitive measures evaluating attention abilities using Pearson correlation.

**Results:**

Theta and alpha activity values were higher in the absence vs the presence of a smartphone, with a significant difference between theta bands for the two study conditions. Moreover, the difference between theta bands in the two conditions was significantly correlated with lower scores on an auditory attention test.

**Conclusions:**

The existence of an interactive electronic device during cognitive tasks is associated with alterations in brain activity related to cognitive control.

**Impact:**

- The presence of a smartphone during a simple reaction time task in young children was associated with a significant decrease in frontal theta frequency.
- A trend of a decreased alpha band in the presence of a smartphone.
- The differences in theta and alpha frequencies between conditions were significantly correlated with lower scores in auditory and visual attention and inhibition tests.

## Introduction

### Screen exposure and child’s brain activation

In the twenty-first century, screens are becoming increasingly involved in our daily lives, especially for children, whether it may be direct exposure or not.^1^ The American Academy of Pediatrics recommended limiting the screen time for children with no screen time until the age of two years and limiting screen exposure (up to 1 h/day) from two years to the age of five years.^2^ Recommendations for older children ages 8–10 years are <2 h of media entertainment per day.^3^ Health concerns due to screen exposure include obesity, sleep disorders, and reduced cognitive, language and social development.^2^ Although smart devices can be beneficial in some cases,^4,5^ their presence also has some negative impacts on child development, which can lead to cognitive and behavioral challenges.^6^ In line with this, parents are recommended to limit their children’s exposure to smart devices and screens in general to reduce these cognitive and behavioral challenges.^7,8^ Additional concerns related to smartphone use in children include health concerns such as sleep problems, weight gain, sugar consumption and behavior disorders, as was found in a study including sixty 4–8-year-old children.^7,8^

In this sequence, technoreference i.e. the usage of mobile screen devices in a way that interferes with parent-child social interactions,^9^ can cause behavioral problems in the child.^10^ A study aiming to reveal the effect of the presence of smart devices on cognitive ability in 21-year-old participants pointed at some interesting findings.^11^ This group was assigned to one of three phone location conditions (distances from the participants): desk, pocket/bag, or other room. Two simple attention tasks were performed in each of the three conditions: the Automated Operation Span task, which requires participants to solve a series of math operations while trying to remember a set of unrelated words,^11^ and the nonverbal task (Raven Matrices), which requires choosing one of six or eight possibilities that appropriately completes the overall series of patterns.^11^ The results suggested that smartphones in the close surrounding environment (even without actual use) could affect cognitive abilities in short and long-term learning abilities.^11^ Similar results were found in a study of 132 18–25-year-old college students who completed a visual working memory task in three conditions: smartphone turned off and visible, smartphone turned on and visible, and smartphone replaced by a calculator.^12^ These two studies suggested that the existence of smartphones while performing cognitive tasks reduced available cognitive resources and significantly affected task performance.^11,12^

The neurobiological correlates for screen exposure were also examined in several age groups and tasks, pointing overall at alterations in brain connectivity and structural integrity. Reduced functional connectivity between the visual word form area associated with word recognition and brain regions related to language and executive functions was related to a longer screen time exposure in 8–12-year-old English speakers.^13^ In the same age group, decreased engagement of executive function networks was observed in typically developing children with increased screen time, which was reversed in situations that involved elevated screen time accompanied by parental dialog.^14^ Younger individuals (3–5 years old children), showed reduced white matter integrity (reduced fractional anisotropy) in several tracts associated with language, literacy, and cognitive control skills in relation to high screen exposure.^15^ Moreover, the brain activity of thirty preschool children aged 4–6 years was divided into two groups: one group listening to stories from a screen vs a group of children listening to stories told by a person for six weeks.^16^ The group who listened to stories on the screen showed significantly higher functional connectivity in theta vs beta bands in regions associated with visual processing and attention abilities, while the person-based storytelling group showed no difference between the bands.^16^ Accordingly, the theta/beta ratio in the group listening to stories on the screen was higher than in the group listening to stories told by a person, which was related to attention load and represented the arousal mechanism.^16^ Another large-scale study conducted among 52 native English-speaking children aged 3–5 years showed that increased screen time, as reported by parental questionnaires, was related to reduced gray matter integrity in the occipital cortex and the prefrontal and lateral cortices.^17^ In addition, it was shown that screen use is linked to lower connectivity between the left visual word form area and language and cognitive control areas.^15^ Taken together, these studies point to the utilization of neural circuits associated with executive functions (or cognitive control) and attention during screen exposure in children. This is especially concerning as childhood through adulthood is the time window for the neurobiological and cognitive development of these abilities.^18,19^

### Cognitive development: the specific case of attention and executive functions

Executive functions (or EF) is an umbrella term that includes higher-order cognitive abilities necessary for learning and achieving a goal.^20^ The “core” EF is related to inhibition (withholding a response), switching (shifting between activities), and working memory abilities,^21^ with suggestions to also include speed of processing.^20^ Neuroimaging studies pointed at neural circuits (networks and cortices) supporting EF even from early infancy during a linguistic task.^22,23^ The development of EF is dependent on the integrity of the connection between frontal cortices and distant regions (occipital) later in development.^18^ The frontal lobe maturation affects the attentional control process, including the capacity to attend to specific stimuli selectively, inhibit prepotent responses, and focus attention for a prolonged period.^20^

One way of characterizing brain activity associated with cognitive control is by using Electroencephalography (or EEG). Waveforms generated by EEG are typically sinusoidal and were classified into five basic frequencies: gamma (30–60 Hz), beta (13–29 Hz), alpha (8–12 Hz), theta (4–7 Hz), and delta (0.5–3 Hz).^24^ Several frequencies were specifically related to distracted performance; theta bands in frontal regions were associated with memory and learning processes; increased theta activity in the prefrontal cortex has been linked to improved working memory.^25^ Theta activity has also been linked to sustained attention and cognitive engagement.^26^ This cognitive engagement is achieved by facilitating information integration from different brain regions, supporting the maintenance and manipulation of information.^27^ Theta band reflects greater attention allocation and cognitive load during working memory tasks.^27^ Moreover, alpha frequencies, specifically in frontal regions, were related to general alerting and ongoing visual processing.^28^ These studies suggest that altered frontal activation manifested by decreased theta or increased alpha powers is related to distracted behavior in adults. Whether a similar phenomenon will be found in children is the topic of the current study.

Despite the accumulated data on the effect of screen exposure on brain structure and functions in children, the neurobiological effects of the presence of smart devices in various levels of proximity while performing a cognitive task are still unknown. Here, brain patterns associated with the presence of smartphones among children performing a simple reaction time task, focusing on frontal theta and alpha frequencies related to distracted performance and cognitive abilities,^25^ will be determined. We hypothesize that decreased theta bands and increased alpha bands will be observed when children perform a simple reaction time task in the presence of a smartphone, compared to a performance in the same task without the existence of smartphones.

## Methods

### Participants

Twenty-three Hebrew-speaking preschool children, ages 5.3–8.5 years (mean age = 6.94, SD = 0.976, 11 males), all native Hebrew speakers, participated in the current study. All the participants were right-handed. None of the participants had neurological or psychiatric disorders or attention difficulties (was assessed using the Conners parental questionnaire^29^). All parents were of an average socioeconomic status. The study was approved by the institutional ethical committee, and written consents were obtained from the parents.

### Study procedure

Participants were invited to the lab at a northern university institution in Israel. They first performed several behavioral/cognitive tests, had a break of approximately 10 min to avoid fatigue and then were invited to the EEG room and performed the simple reaction time task under two conditions: 1) with the presence of a smartphone on the same desk where the computer was located (15 cm distance from the child); 2) without the presence of a smartphone in the room. These two conditions were counterbalanced.

### Behavioral measures

Several behavioral tasks and questionnaires were administered to evaluate EF and attention abilities:

Executive functions: General EF and EF components were assessed using a general EF parental report, which also included switching abilities (Behavioral Rating Inventory Executive Function, BRIEF, parental report).^30^ Working memory was measured using the digit-span task [WPPSI^31^], and shifting skills were measured using the colors-animals inhibition task.^32^ Inhibition abilities were evaluated using the Walk–Don’t-Walk Test from the Test of Everyday Attention (TEA-Ch) battery.^33^ Finally, speed of processing measures (symbol search, coding) from the WPPSI,^31^ were evaluated as well.

Attention abilities were assessed using the visual (Sky search) and auditory attention (Score!) subtests from the TEA-Ch battery.^33^

### Electrophysiological measures

#### EEG data acquisition

While performing the behavioral task, the participant underwent an EEG recording in a soundproof room. The participant sat about 80 cm in front of IBM PC monitor. The data were recorded continuously from 64 electrodes mounted on a custom-made cap (EasyCap, Brain Products GmbH Germany) according to the international 10/20 system. The system used in the current study includes wet electrodes and a conductive gel to enhance electrode conductivity.

**Fig. 1:**
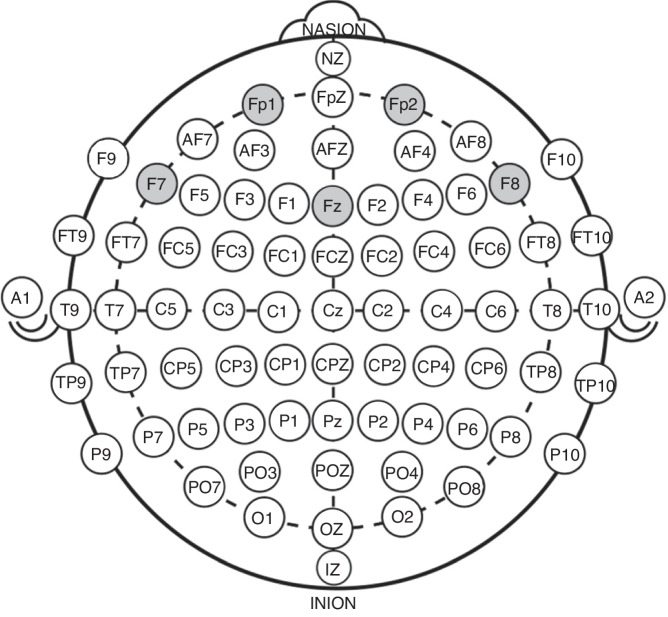
The EEG 64-electrodes distribution. The EEG 64-electrodes distribution. The gray electrodes were used for the analysis.

The data was recorded from the following electrodes: Frontal: FP1, FPz, FP2, AF7, AF3, AFz, AF4, AF8, F7, F5, F3, F1, Fz, F2, F4, F6, F8, FT7, FC5, FC3, FC1, FCZ, FC2, FC4, FC6, FT8; Temporal: T7, T8, TP7, TP8; Central: C5 C3, C1, Cz, C2, C4, C6, CP5, CP3, CP1, CPZ, CP2, CP4, CP6; Parietal: P9, P7, P5, P3, P1, Pz, P2, P4, P6, P8, P10, PO7, PO3, POZ, PO4, PO8; and Occipital: O1, OZ, O2 electrodes, where the TP8 electrode was used as a reference electrode. The ground electrode was placed in a special location at the front of the cap (next to FPz electrode, where the Nz electrode in Fig. 1 is noted). The impedance of all electrodes was kept below 5 KOhm. The data was sampled at a rate of 5000 Hz with an analog bandpass filter of 0.1–70 HZ and 12-bit A/D converter.

### EEG task: the simple reaction time task

**Fig. 2:**
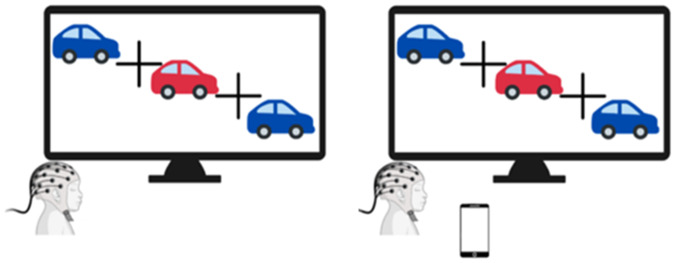
The EEG task design. The EEG task design: The left image represents the condition without a smartphone and the right image represents the other condition with the presence of a smartphone.

During the simple reaction time task, red or blue cars (20 stimuli each), were presented in the center of the screen for 400 msec, with an ISI of 100 msec, representing the time lapse between the alternating stimuli and a response time of 2000 msec ± 200 jitter. The experimenter asked the child to press the left key for the red cars and the right key for the blue cars. To determine the effect of the smartphone’s presence on the child’s brain waves, the task was administered twice in a randomized manner, with and without the presence of a smartphone on the same desk where the computer screen was placed. Each task run lasted 3 min. The average response time and accuracy percentage rates were calculated for each task run. See Fig. 2.

### Data analysis

#### Behavioral data analysis

The Shapiro–Wilk test indicated a normal distribution for all measures used (*P* > 0.05). Several independent one-sample *t*-tests (with an alpha level set to 0.05) were conducted to determine whether the participants’ averaged EF and attention abilities were within the normal ranges based on established age-appropriate standardized norms (BRIEF,^30^ WPPSI,^31^ colors-animals inhibition,^32^ TEA-Ch^33^).

### EEG data analysis

#### Behavioral measures: simple reaction time task

Several paired *t*-tests were conducted to determine the differences in reaction time and accuracy rates in the two task conditions (with and without the presence of a smartphone).

### EEG data preprocessing

After adding the channel locations using a spherical file with eye channels (Standard-10–5-cap385) using the EEGlab MATLAB toolbox, the following preprocessing steps were conducted using the same MATLAB toolbox: re-referencing the data by removing the reference electrode TP8, line noise removal (artifact caused by AC current and affects the gamma band), which was removed using a notch filter at 60 Hz.^34^ Ocular artifacts related to eye movements and blinks^35^ as well as muscle artifacts^35^ were removed using an independent component analysis (ICA) method. In the ICA method, artifacts were rejected by a combination of visual and automatic detection by the algorithm; this step was performed on all subjects’ data, where the number of removed components varied between 1 and 3 for each. In addition, to minimize eye movement artifacts, a cross was placed in the middle of the screen.

**Fig. 3:**
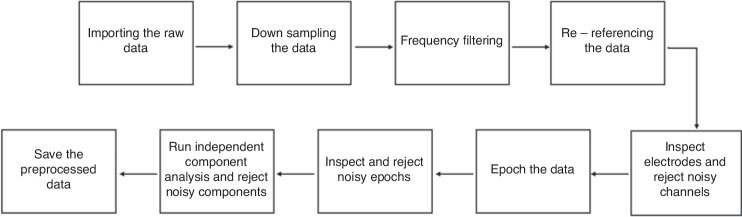
A diagram for the preprocessing procedure. The EEG data preprocessing procedure.

Moreover, several filters were used during the EEG preprocessing steps, including a high Pass Filter (HPF) to minimize signal drifts caused by low-frequency noise, a Low Pass Filter (LPF) to transmit low frequencies and for muscle artifacts removal, and a notch filter to remove AC line voltage noise. In our experiment, data was filtered between 1–45 Hz frequencies. See Fig. 3 for this analysis pipeline.

### Post-processing

Following the preprocessing steps, the data were examined using MATLAB tools to closely investigate changes in frequencies by comparing the frequency spectra of the EEG signal for the two conditions: with and without the phone. Alpha (8–12 Hz) and theta (4–7 Hz) frequencies were extracted from the recorded signal for each participant to assess the differences between the two study conditions. Furthermore, the disparities in theta and alpha frequencies across different conditions were calculated. Averages of theta and alpha power were determined for five frontal electrodes (FP1, Fz, F7, F8, FP2) in both study conditions (see Fig. 1 for the electrode distribution); these averages were calculated by an automatic detection of relevant peaks and ranges using a MATLAB code.

### Statistical analysis EEG data

Paired sample *t*-test analysis was conducted to determine the difference between the conditions within each EEG signal.

To determine the difference in frequencies between the different task conditions, a 2 × 2 Repeated measures ANOVA with Frequency (theta, alpha) and condition (without, with a smartphone) for the averages of the frontal EEG bands was used. Data was corrected for multiple comparisons (corrected significance *p* < 0.0125).

### Correlations between neuroimaging and behavioral measures

To determine the relationship between the different theta and alpha bands in the two conditions and standardized measures for EF and attention abilities, a Pearson correlation was conducted between behavioral results and the difference in frequency bands in the two study conditions (i.e. the existence of a smartphone minus without the existence of a smartphone). The data was corrected for multiple comparisons using a Bonferroni correction.

## Results

### Behavioral results

The behavioral data suggested that all the participants were within the normal range in their EF and attention abilities. See Table 1.

**Table 1:** Executive functions and attention behavioral measures.

Abilities	Measures	M(SD)	Min–max
Executive functions	Inhibition, percentage (BRIEF)	47.26(22.97)	12–88
	Inhibition, percentages (Walk/Don’t walk)	3.652(2.03)	0–7
	Switching Colors (raw)	25.68(4.63)	12–30
	Switching, Animals (raw)	25.10(6.48)	2–30
	Working memory, percentage (BRIEF)	44.66(23.26)	10–86
	Speed of processing, coding subtest, standard score (WIPSSI)	11.21(2.2)	6–16
	Speed of processing, symbol search subtest, standard Score (WIPSSI)	11.73(2.55)	7–16
Attention	Visual attention, Sky search subtest, scale score (TEA-CH)	8.15(3.93)	4–15
	Auditory attention, Score! scale score (TEA-CH)	3.347(1.77)	0–6

### EEG measurements

**Fig. 4:**
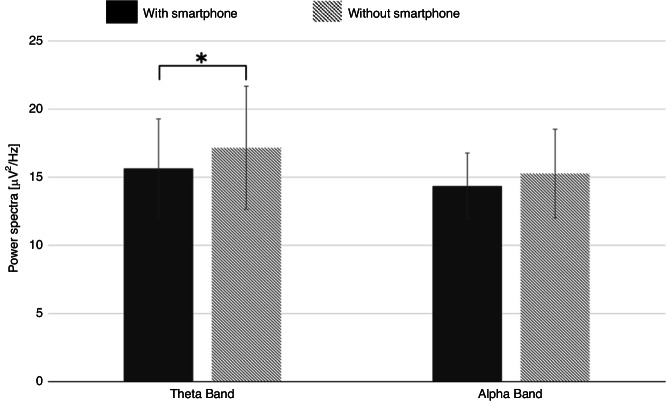
The changes in theta and alpha frequencies in the presence and the absence of smartphones. The changes in theta (left bars) and alpha (right bars) frequencies between two study conditions: with the presence of a smartphone (dark gray) and the absence of a smartphone (light gray). Standard deviations are noted.

Post-hoc paired sample *t*-test analysis for each frequency band for the two conditions revealed a significant decrease for theta frequency bands with decreased power in the presence of smartphones. A similar direction, though not significant, was found for the alpha power. Averages and standard deviations for the theta and alpha bands for the two conditions are noted in Table 2 and in Fig. 4.

**Table 2:** Descriptive statistics and paired sample *t*-test results for the mean spectral power of the different frequency bands in the two study conditions (alpha level = 0.05).

	With the presence of a smartphone		Without the presence of a smartphone		t-test (p)
	Mean [$\frac{{{{{{\rm{\mu }}}}}}{{{{{{\rm{V}}}}}}}^{2}}{{{{{{\rm{Hz}}}}}}}$]	SD[$\frac{{{{{{\rm{\mu }}}}}}{{{{{{\rm{V}}}}}}}^{2}}{{{{{{\rm{Hz}}}}}}}$]	Mean [$\frac{{{{{{\rm{\mu }}}}}}{{{{{{\rm{V}}}}}}}^{2}}{{{{{{\rm{Hz}}}}}}}$]	SD[$\frac{{{{{{\rm{\mu }}}}}}{{{{{{\rm{V}}}}}}}^{2}}{{{{{{\rm{Hz}}}}}}}$]	
Theta band	15.637	3.712	17.160	4.616	−2.104 (0.047)
Alpha band	14.360	2.482	15.267	3.322	−1.538 (0.138)

The results of the repeated measures ANOVA indicated a significant main effect of frequencies (F(1,22) = 19.046, *p* < 0.001, ŋ2 = 0.464), suggesting a significantly greater theta vs alpha activity. Additionally, a trend towards significance for the task’s condition was found (F(1,22) = 3.708, *p* = 0.067, ŋ2 = 0.144), indicating greater alpha and theta frequencies in the absence vs the presence of smartphone while performing the task (alpha band: average 15.267 [(μV^2^)/Hz] vs 14.36 [(μV^2^)/Hz], effect size: 0.309, theta band: average 17.16 [(μV^2^)/Hz] vs 15.637 [(μV^2^)/Hz], effect size: 0.363). No significant interaction effect was found (F(1,22) = 2.546, *p* = 0.125, ŋ2 = 0.104), suggesting that the relationship between frequencies and the task’s condition did not vary significantly.

### Correlations between EEG and behavioral measures

**Fig. 5:**
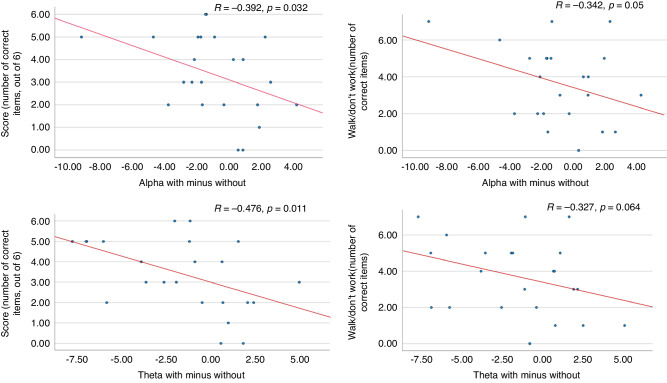
Correlations between the EEG frequencies in the two study’s conditions and the behavioral measures. Correlations between the EEG frequencies (with the presence of smartphone minus the absence of smartphone, x-axis) and the behavioral measure (y-axis). The left graphs are related to auditory attention measures (Score!), and the right graphs are related to an inhibition measure (Walk/Don’t walk). The *r* and *p* values are noted for each graph (significance threshold following correction for multiple comparison; *p* < 0.0125).

Pearson correlations for the difference between theta and alpha frequency bands in the two conditions (with vs without the presence of a smartphone) and EF visual and auditory attention measures revealed significant negative correlations between the difference in theta and alpha frequencies with vs without the smartphone, with measures of auditory and visual attention and inhibition (for the alpha band and auditory attention and inhibition: *r* = −0.392, *p* = 0.032, with visual attention *r* = −0.342, *p* = 0.055; for theta with auditory attention *r* = −0.476, *p* = 0.011, with visual attention *r* = −0.398, *p* = 0.03). Results suggest that the greater the EEG difference between the two conditions- the higher the attention and inhibition scores. As the corrected *p*-value threshold was *p* < 0.0125, only the correlations between auditory attention and theta band (*p* = 0.011) survived the multiple comparisons correction. See Fig. 5 for the regression lines.

## Discussion

The current study aimed to determine the effect of the smartphone’s presence on brain activity during a simple reaction time task in young children. We initially hypothesized that the presence of a smartphone would be associated with lower theta and greater alpha frequency, per previous findings.^25,26,28^ However, our study’s results revealed a different pattern: the existence of a smartphone was related to a significant decrease in frontal theta frequency and a trend of decrease in alpha frequency. Our results will be discussed in light of the cognitive load theory, suggesting that the presence of smartphones can impose additional cognitive demands and hinder task performance by diverting attention and reducing available cognitive resources.

### Decreased frontal theta in the existence of smartphone

The presence of a smartphone during a simple reaction time task in young children was associated with a significant decrease in frontal theta frequency, suggesting a disruption in cognitive control processes. This finding aligns with previous research on attention and cognitive performance, where theta activity was linked to sustained attention and cognitive engagement, i.e. greater theta activity was related to greater attention and cognitive performance.^26^ Moreover, increased theta activity in prefrontal cortex areas was linked to improved working memory in adults aged 24–32 years while performing a visual working memory task.^25^

The observed decrease in frontal theta frequency during smartphone presence may point to a cognitive resource diversion and a shift in cognitive focus, potentially due to higher attentional demands arising from smartphone-related distractions. These higher demands could lead to reduced cognitive efficiency and task performance. Young children, whose cognitive control abilities are still developing,^20^ may be especially vulnerable to the presence of smartphones and the disruption to cognitive skills. These smart devices, which are frequently designed to capture attention, may impair the cognitive processes necessary for task performance, as demonstrated by observed changes – the decrease in frontal theta frequency. This also might impair the children’s cognitive abilities to regulate attention, inhibit distractions and allocate cognitive resources appropriately. Interestingly, this interference still exists even when children are not actively using the device, which might be due to anticipation of messages/notifications received on the device.^36–39^

The significance of comprehending how smartphones affect cognitive functionality during the crucial developmental period is highlighted by this finding and by revealing the alterations in frontal theta frequency due to smartphone interference. Future longitudinal studies should examine if this interference is also observed in older individuals, where EF and attention skills are already well developed, and how different it is from the effect on the developing brain.

### Decreased frontal alpha in the existence of smartphone

The trend of the decreased alpha band in the presence of a smartphone further supports the notion of impaired attentional processes and cognitive inhibition during task performance. This unexpected finding was contrary to our assumptions, as it appears that the presence of smartphones not only diminishes attentional abilities but also impairs cognitive faculties essential for effective task execution. However, previous studies have shown an increased alpha activity during distraction in 20–34 years old individuals while performing a “queuing paradigm” task, which means using symbolic cues like arrows, words, or sounds to guide subjects in switching their attention between tasks or focusing on specific locations, whereas our study focused on preschool children aged 5.3–8.5 years performing a simple reaction time task having simple instructions. This revelation aligns with the prevailing understanding that alpha frequencies, particularly in frontal regions, are linked to the maintenance of overall alertness and the concurrent processing of visual stimuli.^28^ Hence, with the existence of a smartphone, there is a lower-than-optimal activation in this band in frontal regions.

In summary, this unexpected result serves as a turning point in reassessing the multidimensional influence of smartphones on cognitive engagement and load. This work adds to our understanding of the subtle mechanisms impacting cognitive processes in settings involving young children by identifying changes in alpha frequencies within frontal brain areas caused by smartphone-induced interference.

### Correlations between theta frequency and attention abilities

The difference in theta frequency between conditions was significantly correlated with lower auditory attention scores, highlighting the detrimental effects of smartphone interference on cognitive abilities in children. That also aligns with previous research that discussed the link between frontal theta frequencies and task performance.^25,40^

Cognitive control and inhibition are essential for task performance. The disruption in frontal theta frequencies could have implications for children’s ability to inhibit irrelevant stimuli or to control impulsive responses during simple reaction time tasks and may explain the previously reported decreased performance when smartphones are in close proximity to the learning environment in adults.^11^ This can explain the difference in the two frequencies between doing the same task between the two conditions – with vs. without the presence of a smartphone.

The observed decrease in frontal theta and alpha frequencies might result from difficulties with cognitive control processes, leading to reduced efficiency in processing task-relevant and irrelevant information. These results raise concerns regarding the proper ratio of screen time and mental activity during early development and highlight the potentially detrimental effects of smart device use on cognitive function in young children. Additional research is required to identify the precise cognitive control systems impacted by smartphone interference and to consider potential strategies to lessen these effects and support young children’s healthy cognitive development.

### Study’s limitations

The current study includes data from 23 children in a specific age range (early learners). Correction for multiple comparisons resulted in *p* values trending significantly in some cases. While efforts were made to ensure the participants were well-defined and targeted, a larger and more diverse sample could enhance the findings’ generalizability and increase the results’ strength. Secondly, the simple reaction time task used in the study provides evidence of the effect of smartphone existence during basic task performance. However, it might not fully capture the range of cognitive demands encountered during real-world activities. The findings may not extend to more complex cognitive tasks.

## Conclusions

The current study aimed to investigate the effect of smartphone presence on young children’s cognitive processes during a simple reaction time task. Several significant findings emerged through the utilization of EEG measurements and behavioral assessments, shedding light on the intricate relationship between smartphone use and cognitive abilities. This finding underscores the intricate relationship between smartphone presence and cognitive processes. The disruption of alpha frequencies within frontal brain areas further accentuates the potential implications for cognitive engagement and task performance in the presence of smartphones. Furthermore, the correlations between changes in theta and alpha frequencies and measures of attention and inhibition scores highlight the critical importance of understanding the interactions between smartphone use and cognitive functionality during early developmental stages. We advocate a balanced approach to technology use that focuses on limits, age-appropriate material and the development of cognitive abilities to promote children’s learning everywhere. While carefully observing and directing children’s technology use, parents and educators should promote a balanced mix of screen time with other types of learning and play. We can utilize smartphones’ educational potential while preserving children’s cognitive growth by building a healthy technology environment.

## Data Availability

Data will be available upon request (PI: T.H.-K., tzipi.kraus@technion.ac.il).
